# Commentary: The impact of UEFA Euro 2020 football championship on Takotsubo Syndrome: Results of a multicenter national registry

**DOI:** 10.3389/fcvm.2023.1122436

**Published:** 2023-01-26

**Authors:** Francesco Barone-Adesi

**Affiliations:** Department of Translational Medicine, University of Eastern Piedmont, Novara, Italy

**Keywords:** cardiomyopathies, ACS, angina pectoris, Takotsubo, cardiovascular disease

I read with interest the paper of Polimeni et al., where they found an astonishing 11-fold increase in risk of Takotsubo Syndrome (TTS) among the Italian population during the final stage of EURO 2020 football tournament (1). This result seems particularly striking, also considering that a large piece of previous research on overall Acute Cardiovascular Events (ACE) did not find a clear association with watching football (2, 3). However, some methodological issues cast doubts about the accuracy of the results of the present study.

First, it is not clear where rates reported in Tables 2A, 2B come from. Apparently, the authors simply divided the number of specific types of ACE by the total number of hospital admissions. However, this procedure would be wrong as, by definition, a rate is the number of cases divided by the person-time of observation.

Second, it is not clear why rates for the control period are different in Tables 2A, 2B, as the same reference was used along the whole paper.

Third, Table 2B shows that rates of TTS on semifinal/final matches and control period were 0.07 and 0.01, respectively. Thus, the crude Rate Ratio (RR) would be expected to be 7, not 11. This issue does not seem due to rounding problems, as the authors used 5-digit numbers in the table when needed. Is this an adjusted RR? If yes, the authors should specify for which variables they have controlled for and possibly providing the crude estimate as well.

Forth, the result of the authors seems also inconsistent with the RR that can be calculated from Table 4 using the reported absolute numbers of cases, namely 10 during the semifinal/final matches and 13 during the control period. The authors stated that the effect of matches was analyzed on the day that the match was played and the day after. Thus, the observation time was 4 days for the semifinal/final stage, while the control period was reported by the authors being of 20 days. As the catchment area of the hospitals (and hence the source population) did not change during the tournament, these figures would correspond to a RR of 3.85 [i.e., (10/4): (13/20)], which is again very different from the reported result. It would be good if the authors could provide the absolute numbers on which their calculations are based.

As a final remark, one could even ask whether an 11-fold increase in TTS after watching a football match is plausible, considering that it is averaged over a 48-h period, whereas triggers typically increase cardiovascular risk over a short time period (2). Assuming that the risk is increased within the first 2 h from the start of a match, the RR of 11 over a 48-h period found by Polimeni would correspond to an acute relative risk of 240 (i.e., an RR of 11 is the 48-h average between an RR of 1.00 during 46 h and an RR of 240 during 2 h). Moreover, only a proportion of the population watches the matches (and is thus exposed). Assuming a proportion of viewers of 50%, the corresponding acute RR among viewers would be 480. To put this number in context, it is worthy to consider that many individually based, case-crossover studies suggest that most triggers increase the cardiovascular risk of 2–7 times in the 1–2 h following the exposure, with the notable exception of cocaine consumption that entails and acute increase in risk of about 20 times (2). This obvious inconsistency with the effects of well-known cardiovascular triggers should suffice to warrant extra-caution in interpreting the results of this study.

## Author contributions

The author confirms being the sole contributor of this work and has approved it for publication.
